# Prognostic genomic alterations in patients undergoing liver resection for hepatocellular carcinoma

**DOI:** 10.1007/s11033-024-09396-7

**Published:** 2024-03-27

**Authors:** SB Nordkild, LB Ahlborn, CW Yde, JM Kugler, J. Klubien, D. Akdag, GL Willemoe, SD Nielsen, Hans-Christian Pommergaard

**Affiliations:** 1 Department of Surgery and Transplantation, Copenhagen University Hospital, Rigshospitalet Inge Lehmanns Vej 7, Copenhagen Ø, 2100 Denmark; 2 Center for Genomic Medicine, Copenhagen University Hospital, Rigshospitalet, Denmark; 3 Institute for Molecular and Cellular Medicine, University of Copenhagen, Panum Institute, Copenhagen, Denmark; 4 Department of Pathology, Copenhagen University Hospital, Rigshospitalet, Denmark; 5 Viro-immunology Research Unit, Department of Infectious Diseases, Rigshospitalet, University of Copenhagen, Rigshospitalet, Denmark; 6 Institute for Clinical Medicine, University of Copenhagen, Panum Institute, Copenhagen, Denmark

**Keywords:** Hepatocellular carcinoma, HCC, MYC amplification, ARID1A, Biomarker, Prognosis

## Abstract

**Introduction:**

Genetic mutations and amplifications found in hepatocellular carcinoma (HCC) have a potentially prognostic impact. The aim of this study was to investigate the prognostic value of mutations and amplifications in HCC from patients that were liver resected.

**Methods:**

Patients liver resected for HCC at Copenhagen University Hospital Rigshospitalet between May 2014 and January 2018 were included. DNA from freshly frozen tumour tissue was investigated with TruSight Oncology 500. Mutations and amplifications were correlated with disease-free survival and overall survival using multivariate Cox regression to assess the effect on prognosis.

**Results:**

Of the 51 patients included, 88% were male and the median age was 69 years. Most patients had a single tumour (84%) with no vascular invasion (67%) in a non-cirrhotic liver (76% with fibrosis, 24% with cirrhosis). The median follow-up was 37 months. Patients with a *MYC* amplification (8%) were significantly younger than the remaining patients. Furthermore, they had a significantly shorter overall survival (15 months (95% CI: 0.0–31.6) vs. 59 months (95% CI: 34.4–83.6), *p* = < 0.001) and disease-free survival (8 months (95% CI: 4.6–11.4) vs. 19 months (95% CI: 12.3–25.7), *p* = 0.03). However, only overall survival remained statistically significant in the adjusted analysis. Furthermore, all patients with an *ARID1A* mutation (6%) had microvascular invasion and significantly larger tumours than the patients without *ARID1A* mutation.

**Conclusion:**

*MYC* amplifications had a prognostic influence on survival, whereas *ARID1A* gene mutations were correlated with microvascular invasion. These may serve as prognostic biomarkers and should be validated in large, independent cohort.

## Introduction

Hepatocellular carcinoma (HCC) is the sixth most common cancer in the world and a leading cause of cancer-related mortality worldwide [1]. Chronic hepatitis B and C virus infections are common in HCC patients, especially in Asia and Africa, and are drivers of HCC development and progression. Other important aetiologies, especially in Western countries, include non-alcoholic steatohepatitis and chronic alcohol abuse leading to liver cirrhosis [2].

HCC is an aggressive cancer with a high mortality rate in advanced stages. However, in early stages, patients may benefit from curative treatments, such as liver resection, ablation, or liver transplantation [2–4]. The decision of a treatment strategy is primarily based on tumour burden, liver function, and patient performance status [2]. Resection retains sufficient liver function and is typically the first choice for HCC patients with a single tumour. However, as many as 70% of the resected patients develop recurrence within five years [2, 5].

Key drivers in the malignant development of HCC are a number of genetic alterations (often (in)activating mutations or gene amplifications). Among the genes frequently altered in HCC patients are *TERT*, *TP53, MYC* amplification, *CTNNB1*, and *ARID1A*, which occur in up to 60%, 48%, 33%, 30%, and 17% of cases, respectively [6–8]. Several genetic alterations in these genes have been associated with a negative impact on survival. In previous studies, *MYC* amplification was found to be associated with larger, more aggressive tumours in patients who are often younger. *ARID1A* was associated with carcinogenesis and metastasis in already established HCC tumours [7, 9]. Notably, few prognostic biomarkers are available today for a personalized HCC treatment approach allowing to stratify patients in the groups that would benefit the most from surgical treatment and other groups where less aggressive treatment would be preferable [4].

Most previous studies on genetic alterations in HCC have been conducted on patients of Asian, American, or Southern European descent with a high prevalence of cirrhosis as well as chronic viral hepatitis C infection [7, 10–14]. The association between clinically significant genetic alterations and prognosis have yet to be described in a population with a low prevalence of cirrhosis and viral hepatitis undergoing resection for HCC.

The aim of this study was to investigate genetic alterations as prognostic markers in a Danish population of patients with HCC with a low prevalence of cirrhosis and viral hepatitis C undergoing liver resection.

## Materials and methods

This study was a retrospective cohort study including all patients that were liver resected for HCC at Copenhagen University Hospital Rigshospitalet, Denmark, between May 2014 and January 2018, with available freshly frozen tumour tissue stored in The Danish Cancer Biobank. The study was approved by the regional ethics committee (journal-nr.: H-18,015,944).

The collected tumour tissue was investigated for genomic changes through next generation sequencing with the TruSight Oncology (TSO) 500 Assay of 523 genes for DNA variants. DNA was extracted from fresh frozen tumour samples using AllPrep DNA/RNA/protein Extraction Kit (Qiagen) according to manufacturer’s instructions. Library preparation was done using TSO500 solid kit from Illumina according to Illumina’s reference guide. Libraries were sequencing using 2 × 150 bp paired-end sequencing on Illumina NovaSeq6000 platform. Reads were aligned to the human reference genome (hg19/GRCh37) and gene variants and amplifications were called using the TSO500 Local App pipeline for data processing (Illumina). Further filtering and identification of cancer-associated mutations (variants classified as pathogenic or likely pathogenic) was performed using Qiagen Clinical Insight (QCI) software from Qiagen.

The following characteristics of the patients, pathology data, and follow-up-data were retrieved from electronic medical records: patient characteristics (age, sex, comorbidities including cirrhosis, and medical history), tumour characteristics (tumour size, number of tumours, microvascular invasion, and resection margins), and follow-up including recurrence, disease-free survival (defined as the time from date of liver resection to the date of detected recurrence), and overall survival (defined as the time from date of liver resection to the date of death). Tumour-stage of HCC was determined according to the AJCC 7th edition criteria [15]. A free margin in the pathological specimen was defined as 1 mm or more between tumour and resection margin. To determine fibrosis/cirrhosis in non-tumour liver tissue, a METAVIR score was evaluated by a specialized pathologist [16].

Patients were preoperatively assessed radiologically and clinically using the Barcelona Clinic Liver Cancer staging and discussed at multidisciplinary team conference. The diagnosis was confirmed in approximately one third of the cases by a preoperative needle biopsy. This is usually an option in non-cirrhotic patients with suspected HCC. Liver function was assessed using the Child-Pugh score. Portal hypertension was assessed with imaging (e.g. collaterals, splenomegaly, re-canalization of the umbilical vein, and ascites) and thrombocyte count. In cases with suspicion of impaired liver function or cases with large resections, ICG-clearance was performed. Patients with impaired liver function were generally not considered for resection and was treated according to the Barcelona Clinic Liver Cancer staging system [17].

As part of clinical practice, patients were followed with abdominal Computed Tomography-scans 3, 6, 9, 12, 18, 24, 36, 48, and 60 months after resection to identify potential recurrence.

### Statistical analyses

The patients were divided into groups according to the genetic alterations detected by the TSO 500 assay. Association between groups of interest and prognosis (disease-free survival and overall survival) was determined by Kaplan Meier statistics reported as median survival with 95% confidence intervals (CI). Log-rank test was used to compare the survival of the different groups. In multivariate Cox-regressions, the association was adjusted for possible confounders. Of the possible confounders (male sex, age, size of HCC, number of tumours, cirrhosis, microvascular invasion, and resection margins), variables significantly associated with the outcome in univariate analyses were included in the multivariate analysis. Moreover, the association between the groups and markers of tumour biology (microvascular invasion, tumour grade, and size) was investigated with Fisher’s exact test. Continuous variables are reported as median with range or interquartile range. The Mann-Whitney Test was used to compare the median age of the patients as well as median size of the largest tumour in patients with and without *MYC* amplification as well as mutations affecting *ARID1A*. The reverse Kaplan-Meier method was used to determine the median follow-up. Follow-up was defined as the median time from the date of liver resection to either event (death) or last follow-up (03.11.2022).

An a priori sample size calculation was based on an earlier study in patients with HCC, where a 5-Gene Score was associated with inferior five-year disease-free survival (78% vs. 33%) [18]. Based on an alpha of 5% and a power of 80%, we needed 34 patients in the study to be able to detect a difference of this magnitude (G-power, version 3.1.9.3.).

We used SPSS, version 23 (IBM Corp, Armonk, NY, USA) to conduct the statistical analysis. The significance level was adjusted to the number of analyses of gene groups using the Bonferroni correction setting the significance level to 0.005.

## Results

### Patient characteristics

In total, 117 patients were liver resected for HCC at Copenhagen University Hospital Rigshospitalet, Copenhagen between May 2014 and January 2018. We identified 54 patients with tumour tissue as well as non-tumorous adjacent tissue stored as freshly frozen tissue in the Danish cancer biobank. Patients with freshly frozen tissue were primarily from the later part of the period where storage of freshly frozen tissue was part of the standard treatment. Of these 54 patients, one was excluded due to liver transplantation as the surgical choice of treatment. Another patient died 41 days after resection due to liver failure, probably caused by the surgery. Unfortunately, the patients’ genetic characteristics were not determined as the patient was excluded prior to analyses. Lastly, one patient appeared twice in the biobank, leaving 51 patients available for analyses. The median follow-up was 37 months (31–41).

Patient characteristics are shown in Table 1. Most patients were male (88%), had a median age of 69 years, a pT1 stage tumour, a median tumour size of 45 mm (largest tumour), and a single tumour without microvascular invasion which were resected with free resection margins. The majority had fibrosis (METAVIR stage 1–3) at time of the resection. However, 12 patients (24%) had cirrhosis (METAVIR stage 4). Complications occurred in 5 (9.8%) of the 51 patients including two cases of fascial dehiscence (3.9%), two cases of bile leakage (3.9%), and one case of pulmonary embolism (2%). Furthermore, 42 patients (82.4%) had recurrence during the study period with a median time to recurrence of 14 months.

**Table 1:** Patient characteristics

	All patients, *n* = 51		Patients w *MYC*, *n* = 4		Patients w/o *MYC*, *n* = 47
Sex, n (%)					
Women	6 (12)		0		6
Men	45 (88)		4		41
Age, years, median (range)	69 (23–88)		58 (50–69)		80 (23–88)
Liver disease, n (%)					
Alcoholic cirrhosis Hepatitis C virus Hemochromatosis Non-alcoholic steatohepatitis No known liver disease	8 (16) 10 (20) 2 (4) 1 (2) 30 (59)		2 1 0 0 1		6 9 2 1 29
Stage, n (%)					
pT1	24 (47)		1		23
pT1b	4 (8)		2		2
pT2	9 (18)		0		9
pT2a	1 (2)		0		1
pT3	1 (2)		0		1
pT3a	8 (16)		1		7
pT3b	3 (6)		0		3
pT4	1 (2)		0		1
Number of tumours, n (%)					
1	43 (84)		4		39
2	6 (12)		0		6
3	2 (4)		0		2
Free resection margins, n (%)					
Yes	41 (80)		2		39
No	10 (20)		2		8
Diameter of the largest tumour, mm, median (range)	45 (17–250)		75 (30–85)		45 (17–250)
METAVIR, n (%)					
Stage 1–3	39 (76)		3		36
Stage 4 (cirrhosis)	12 (24)		1		11
Vascular invasion, n (%)					
Yes	17 (33)		0		17
No	34 (67)		4		30
MELD, median (IQR)*	28.9 (27.3–29.7)		NA		NA
BCLC					
Very early stage (0) Early stage (A) Intermediate stage (B)	3 40 8		0 4 0		3 36 8

* 1 of 4 patients with a MYC amplification and 22 of 51 patients in total had a MELD-score available. These are therefore displayed as “NA”.

HR, hazard ratio; CI, confidence interval; METAVIR, Meta-analysis of Histological Data in Viral Hepatitis; MELD, Model for End-Stage Liver Disease; BCLC, Barcelona Clinic Liver Cancer

### DNA sequencing

The ten most frequently occurring genomic alterations within the study group are shown in Table 2. Mutations affecting *TERT*, *CTNNB1*, and *TP53* were the most frequent genetic alterations and were observed in 51%, 31%, and 31% of the patients, respectively. The most frequent amplification affected the *CCDN1* gene, appearing in 10% of the patients. *MYC* amplification occurred in 8% of the patients.

**Table 2:** Frequently occurring mutations and amplifications

Frequent genomic mutations and amplifications	
*TERT*, n (%)	26 (51)
*CTNNB1*, n (%)	16 (31)
*TP53*, n (%)	16 (31)
*ATM*, n (%)	5 (10)
*FGF3*, n (%)	5 (10)
*FGF19*, n (%)	5 (10)
*FGF4*, n (%)	5 (10)
*ARID1A*, n (%)	3 (6)
*CCND1* amplification, n (%)	5 (10)
*MYC* amplification, n (%)	4 (8)

Of note, the median age of patients with *MYC* amplification was significantly lower (58 years (50–69)) than the remaining patients (69 years (23–88), *p* = 0.018). The median diameter of the largest tumour was 75 mm in patients with *MYC* amplification and 45 mm in the remaining patients (*p* = 0.754).

### Survival analysis

**Fig. 1:**
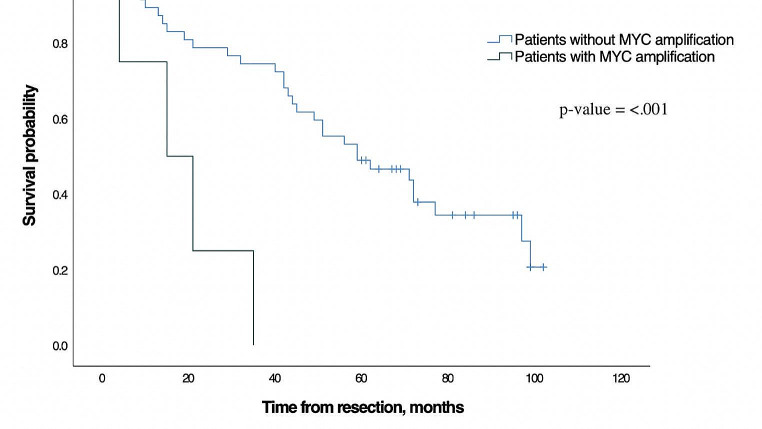
Overall survival for patients without *MYC* amplification and for patients with *MYC* amplification

The median overall survival of all the patients in this study was 56 months (95% CI: 39.2–72.8). For all patients without *MYC* amplification the median overall survival was 59 months (95% CI: 34.4–83.6) compared to 15 months in patients with *MYC* amplification (95% CI: 0.0–31.6), *p* = < 0.001, Fig. 1). Among possible confounders (male sex, age, size of HCC, number of tumours, cirrhosis, microvascular invasion, and resection margins) only male sex and age were significantly associated with overall survival in the univariate Cox-regression (Table 3). Thus, these variables were included in the multivariate analysis with *MYC* amplification. *MYC* amplification remained independently associated with inferior overall survival when adjusted for male sex and age (p = < 0.001, HR: 8.6, 95% CI: 2.5–30.3), and thus remained significant when corrected for multiple comparison with Bonferroni correction. Furthermore, male sex and age were independently associated with inferior overall survival (Table 4).

**Table 3:** Univariate Cox regression analysis

	DFS			OS		
Variable	HR	95% CI	*p*-value	HR	95% CI	*p*-value
*TERT*	1.78	0.96–3.32	0.07	1.76	0.88–3.52	0.10
*CTNNB1*	1.17	0.61–2.25	0.64	1.35	0.67–2.72	0.40
*TP53*	0.72	0.36–1.44	0.36	0.68	0.32–1.43	0.31
*ATM*	0.76	0.27–2.12	0.59	0.67	0.23–1.93	0.46
*FGF3*	1.23	0.44–3.47	0.69	0.94	0.33–2.70	0.91
*FGF19*	1.23	0.44–3.47	0.69	0.94	0.33–2.70	0.91
*FGF4*	1.23	0.44–3.47	0.69	0.94	0.33–2.70	0.91
*ARID1A*	0.79	0.19–3.27	0.74	0.65	0.15–2.76	0.56
*CCND1* amplification	1.23	0.44–3.47	0.69	0.94	0.33–2.70	0.91
*MYC* amplification	3.29	1.10–9.83	**0.03**	5.66	1.80-17.84	**0.003**
Male sex	14.22	1.91-105.97	**0.01**	11.10	1.42–86.74	**0.02**
Age	1.02	0.99–1.05	0.23	1.05	1.00-1.09	**0.04**
Number of tumours	1.39	0.80–2.40	0.24	1.31	0.72–2.40	0.38
Free resection margins	0.73	0.33–1.58	0.41	0.66	0.29–1.54	0.34
Diameter of the largest tumour	0.99	0.99-1.00	0.68	1.00	0.99-1.00	0.91
METAVIR	1.24	0.60–2.53	0.56	1.69	0.80–3.55	0.17
Microvascular invasion	1.34	0.70–2.57	0.38	1.05	0.51–2.12	0.90

HR, hazard ratio; CI, confidence interval; DFS, disease-free survival; OS, overall survival; METAVIR, Meta-analysis of Histological Data in Viral Hepatitis; Bold, statistically significant association (before correction)

**Table 4:** Multivariate Cox regression analysis with overall survival

	HR (95% CI)	*p*-value
*MYC* amplification	8.6 (2.5–30.3)	< 0.001
Age	1.1 (1.0–1.1)	0.013
Male sex	5.7 (1.2–26.8)	0.029

HR, hazard ratio; CI, confidence interval

**Fig. 2:**
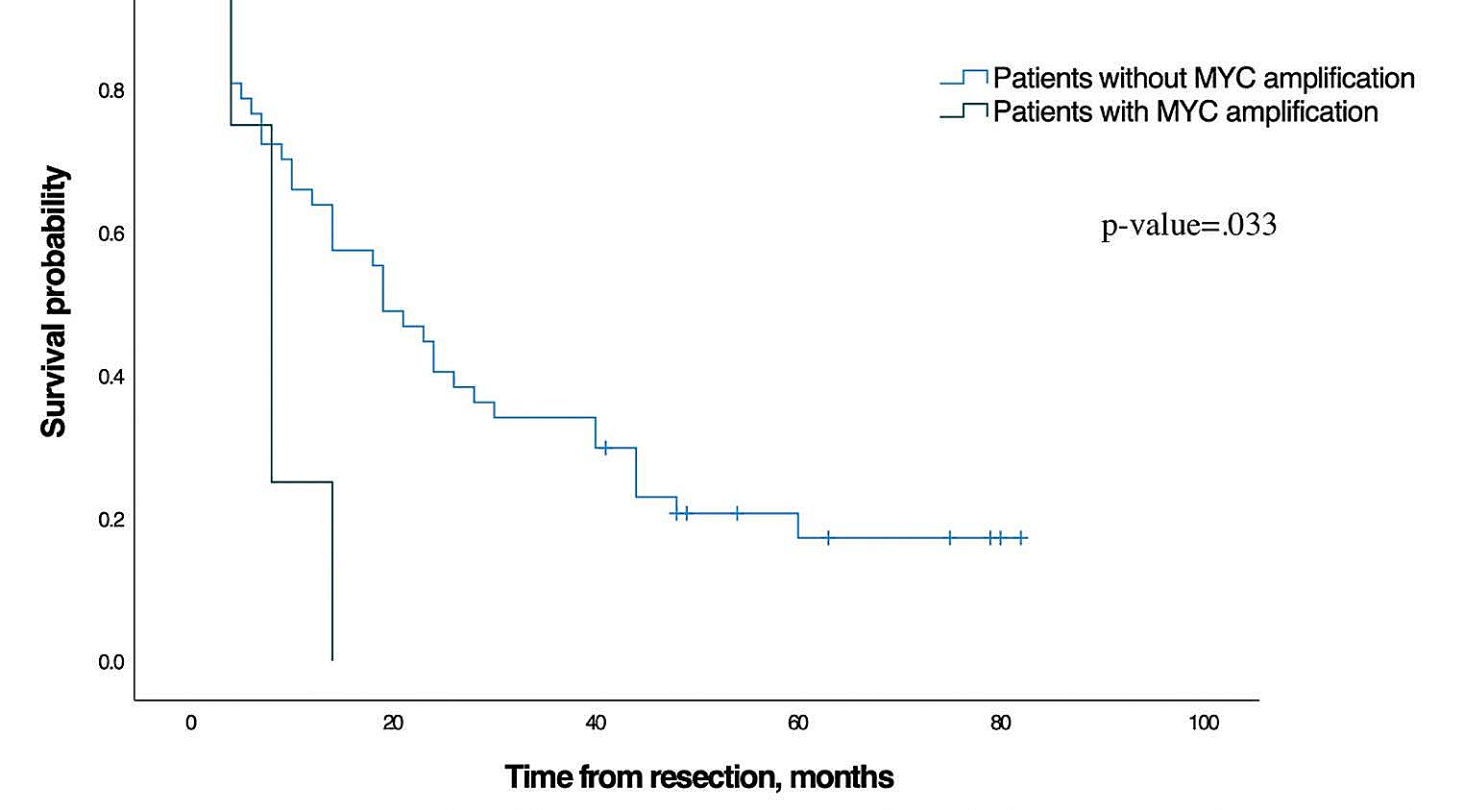
Disease-free survival for patients without *MYC* amplification and for patients with *MYC* amplification

The disease-free survival was shorter in patients with *MYC* amplification (8 months (95% CI: 4.6–11.4)) compared with the remaining patients (19 months (95% CI: 12.3–25.7), *p* = 0.03, Fig. 2). Among possible confounders (male sex, age, size of HCC, number of tumours, cirrhosis, microvascular invasion, and resection margins) only male sex was significantly associated with disease-free survival in the univariate Cox-regression and, thus, included in the multivariate analysis (Tables 3 and 5). *MYC* amplification was not associated with microvascular invasion, tumour grade, or size.

**Table 5:** Multivariate Cox regression analysis with disease-free survival

	HR (95% CI)	*p*-value
*MYC* amplification	2.8 (0.9–8.4)	0.064
Male sex	13.8 (1.8–102.8)	0.011

HR, hazard ratio; CI, confidence interval

The remaining mutations and amplifications listed in Table 2 were not associated with overall or disease-free survival.

### Markers of tumour biology

We found a larger tumour size in patients with microvascular invasion (median 85 mm) compared to patients without (median 41 mm, *p* = 0.03). All patients with a mutation in the *ARID1A* gene had microvascular invasion compared with only 29.2% of patients without the mutation (*p* = 0.03). However, *ARID1A* was not associated with disease-free survival, overall survival, tumour grade, or size.

The remaining mutations and amplifications listed in Table 2 were not significantly associated with tumour grade, size, or microvascular invasion. Furthermore, a correlation between aetiology and mutations could not be determined.

## Discussion

In patients that were liver resected for HCC, we showed that *MYC* amplification was associated with a reduced overall survival. The association with overall survival remained significant when adjusted for male sex and age. The study also demonstrated that mutations in the gene *ARID1A* were associated with microvascular invasion in the tumour, and that patients with microvascular invasion had significantly larger tumours than patients without.

The association between the *MYC* amplification and a poorer prognosis has previously been shown in American, Italian, and Japanese studies [7, 10, 11]. All three studies found that *MYC* amplification was more frequent among HCC patients with high-grade tumours and found a higher prevalence of *MYC* amplification (19%, 33%, and 50%) compared with the 8% in our study. The reason for the variation in the frequency of *MYC* amplification may be due to different aetiologies for HCC between the studies. Only 24% of the patients included in the present study had cirrhosis at time of resection which is considerably lower than the 78.5% of the patients in the Italian study [7]. Moreover, the prevalence of hepatitis C virus in our study population was only 20% compared to 67% in the Japanese study [11]. In accordance with our results, the amplification was primarily found in younger patients (age < 50 years) with larger tumours in the Japanese study.

The clinical significance of mutations in *ARID1A* in HCC is still debated. One study reported that a loss of *ARID1A* was associated with a poorer prognosis and that *ARID1A* may exhibit a tumour suppressive role. The same study found that *ARID1A* is not carcinogenic alone but rather accelerates the carcinogenic process when associated with other oncogenes such as an amplification of *MYC* [8]. Another study showed that *ARID1A* had a context dependent role: overexpression of *ARID1A* was linked to the initiation of HCC, whereas a loss of *ARID1A* was linked to further metastasizing of a primary HCC [9]. We found that a mutation in the gene was associated with vascular invasion which is known to be a predictor of a poor prognosis [19]. However, *ARID1A* was not in itself prognostic in our study, which may be a result of the limited sample size and/or different aetiologies.

The present study adds the current evidence in the field by investigating the impact of *MYC* amplification and *ARID1A* on prognosis in a population of patients with a low prevalence of cirrhosis and viral hepatitis undergoing liver resection for HCC. Moreover, we evaluated HCC using the TSO 500 assay, which includes a wide range of oncogenes selected due to previously findings of prognostic significance. Our results corroborate with findings of previous studies, which emphasises the apparent clinical significance of *MYC* amplification. The patients in this study represent a homogenous group with long follow-up and high event rates. We conceived this work as an explorative study, using a limited number of available patient samples with detailed follow-up. This, however, is a limitation of this study. We were unable to adjust for potential confounders known to impact survival in a multivariate analysis, thus only show an association between *MYC* amplification and survival. Given the small sample size it is important to interpret these findings with caution and acknowledge the reduced generalizability. Therefore, future studies with a larger sample size are warranted, which could underline these results and possibly detect further relevant associations using the TSO 500 approach. This will enable adjustment for potential confounders and strengthen the results.

Few, biomarkers are used clinically in the treatment of HCC today. With a high recurrence rate as shown in the present study, biomarkers for personalized treatment are needed. Preoperative *MYC* amplification may have a strong prognostic impact but cannot be determined without a preoperative needle biopsy from the tumour. A preoperative needle biopsy bears a certain risk of tumour seeding to surrounding organs (2.7%) and structures and risk of bleeding [20]. Hence, we consider it of interest to further evaluate prospectively whether the knowledge of candidate biomarker status, such as *MYC* amplification, outweighs the potential negative effects risked by taking a preoperative tumour biopsy.

In conclusion, we showed that *MYC* amplification was associated with a poorer prognosis. Furthermore, we showed that a mutation in *ARID1A* was associated with vascular invasion. After validation in a prospective setup, the findings of the present study have the potential to affect surgical treatment strategy for patients with HCC.

## Data Availability

The Danish Data Protection Agency does not allow open access to the data included in this study. However, reasonable requests for additional analyses on the dataset can be made to the corresponding author.
